# Abdominal fat pad biopsies exhibit good diagnostic accuracy in patients with suspected transthyretin amyloidosis

**DOI:** 10.1186/s13023-020-01565-8

**Published:** 2020-10-08

**Authors:** Hedvig Paulsson Rokke, Nima Sadat Gousheh, Per Westermark, Ole B. Suhr, Intissar Anan, Elisabet Ihse, Björn Pilebro, Jonas Wixner

**Affiliations:** 1 Department of Public Health and Clinical Medicine, Umeå University, 901 87 Umeå, Sweden; 2 Department of Immunology, Genetics and Pathology, Uppsala University, Uppsala, Sweden; 3 Wallenberg Centre for Molecular Medicine, Umeå University, Umeå, Sweden

**Keywords:** Adipose tissue, Amyloid polyneuropathy, Amyloidosis, hereditary, Biopsy, Cardiomyopathy, restrictive, Techniques and procedures, diagnostic, Transthyretin

## Abstract

**Background:**

The diagnostic accuracy of histopathological detection of transthyretin amyloid (ATTR) by Congo red staining of abdominal fat samples has been questioned since low sensitivity has been reported, especially for patients with ATTR cardiomyopathy. However, the outcome of surgically obtained fat pad biopsies has not yet been evaluated. The aim was to evaluate the diagnostic accuracy of skin punch biopsies from abdominal fat in patients with suspected ATTR amyloidosis.

**Material and methods:**

Data were evaluated from patients who had undergone abdominal fat pad biopsies using a skin punch due to suspected amyloidosis from 2006 to 2015. The biopsies had been analysed using Congo red staining to determine the presence of amyloid, and immunohistochemistry or Western blot to determine the type of amyloidosis. The final diagnosis was based on the clinical picture, biopsy results and DNA sequencing. Minimum follow-up after the initial biopsy was 3 years.

**Results:**

Two hundred seventy-four patients (61% males) were identified, and in 132 (48%), a final diagnosis of amyloidosis had been settled. The majority (93%) had been diagnosed with hereditary transthyretin (ATTRv) amyloidosis, and therefore subsequent analyses were focused on these patients. Overall, our data showed a test specificity of 99% and a sensitivity of 91%. Ninety-eight (94%) of the patients had neuropathic symptoms at diagnosis, whereas 57 (55%) had signs of amyloid cardiomyopathy. Subgroup analyses showed that patients with merely neuropathic symptoms displayed the highest test sensitivity of 91%, whereas patients with pure cardiomyopathy displayed the lowest sensitivity of 83%. However, no significant differences in sensitivity were found between patients with or without cardiomyopathy or between the sexes.

**Conclusions:**

Abdominal fat pad biopsies exhibit good diagnostic accuracy in patients with suspect ATTRv amyloidosis, including patients presenting with cardiomyopathy. In addition, the method enables typing not only of the precursor protein but also of the amyloid fibril type, which is related to the phenotype and to the outcome of the disease.

## Background

Amyloidosis is an acquired or inherited condition caused by extracellular deposition of insoluble fibrillar protein (amyloid) that is formed by misfolded proteins that assemble into beta-pleated fibrils. Amyloid formation and deposition can be localized or systemic, and can negatively affect the function of the targeted tissues [1]. Demonstration of amyloid deposits in tissue samples using Congo red staining and polarized light microscopy is the gold standard for diagnosing amyloidosis [2]. Tissue samples can be obtained either from the primarily affected organ (e.g. heart or peripheral nerves), or in the case of systemic amyloidosis, from more easily accessible tissues (e.g. abdominal fat, labial salivary glands or the digestive tract) [3]. The tissue of choice varies between clinics depending on local traditions and access to diagnostic procedures. Typing of the amyloid precursor protein is vital since it has implications on disease pathogenesis and on the choice of therapy [3–5]; this is usually performed by immunohistochemistry, immune electron microscopy, mass spectrometry or Western blot analysis.

Transthyretin (ATTR) amyloidosis is one of the major systemic amyloidoses and is induced by the deposition of misfolded transthyretin (TTR). Hereditary transthyretin (ATTRv) amyloidosis is caused by *TTR* mutations that decrease the stability of the TTR tetramer, whereas acquired (wild-type) transthyretin (ATTRwt) amyloidosis is related to tetramer destabilization with increasing age and possibly also to a decreased protein aggregate clearance in the elderly [6, 7]. ATTRwt amyloidosis is being increasingly recognized and may affect up to 25% of individuals over the age of 80 years according to post-mortem studies [8, 9]. Although rare, ATTRv amyloidosis is the most widespread inherited systemic amyloidosis that can be found all over the world with certain clustering areas in, for example, Portugal, Brazil, Mallorca, Sweden and Japan [10, 11]. To date, over 120 amyloidogenic *TTR* mutations have been demonstrated of which the *TTR* V30M (*p. V50M*) is the most common variant [11, 12]. The disease phenotype is related to the genotype; however, phenotypic variations have also been observed within genotypes. Most genotypes are characterized by neuropathic complications with or without cardiac involvement, although some genotypes are associated with mainly oculoleptomeningeal or cardiac complications [10]. In ATTR amyloidosis, the amyloid fibril type has also been shown to be associated with the disease phenotype. Amyloid deposits containing either a mixture of TTR fragments and full-length TTR (Type A) or full-length TTR only (Type B) have been noted. In ATTRV30M amyloidosis, type A fibrils are generally associated with older age at onset and amyloid cardiomyopathy, whereas type B fibrils are associated with younger age at onset and mainly autonomic and peripheral neuropathic complications [13]. Moreover, the presence of type A fibrils has been linked to a poorer outcome after liver transplantation, which until quite recently has been the only available treatment for ATTRv amyloidosis [14].

In Sweden, we have a long tradition of performing abdominal fat pad biopsies for diagnosing systemic amyloidosis in general and for ATTR amyloidosis in particular [15]. Further, surgically obtained abdominal fat pad biopsies have been the standard diagnostic method at the Amyloidosis Centre in Umeå since 2006. Our clinical experience is that this is a safe and reliable method. However, the diagnostic accuracy of abdominal fat pad biopsies has been questioned in patients with ATTR cardiomyopathy, especially in regard to fine needle aspirates [16, 17]. Recently, it was suggested that bone scintigraphy (e.g. ^99m^Tc-DPD scintigraphy) is a reliable and safe non-invasive method for diagnosing ATTR cardiomyopathy [18]. Therefore, the aim of the current study was to evaluate the diagnostic accuracy of abdominal fat pad biopsies in patients with suspected ATTR amyloidosis, and to explore possible differences in diagnostic specificity and sensitivity across phenotypes.

## Methods

Data were evaluated from all patients who had undergone at least one abdominal fat pad biopsy at the Amyloidosis Centre, Umeå University Hospital between January 2006 and December 2015. This time span was chosen since electronic medical records were introduced at our centre in 2006 and since a follow-up time of at least three years was considered to be sufficient to capture any possibly false negative results. The code TQX10 (punch biopsy) from the Swedish Classification of Health Interventions (KVÅ) version 2019 was used to identify the patients in our medical records in January 2019.

**Fig. 1:**
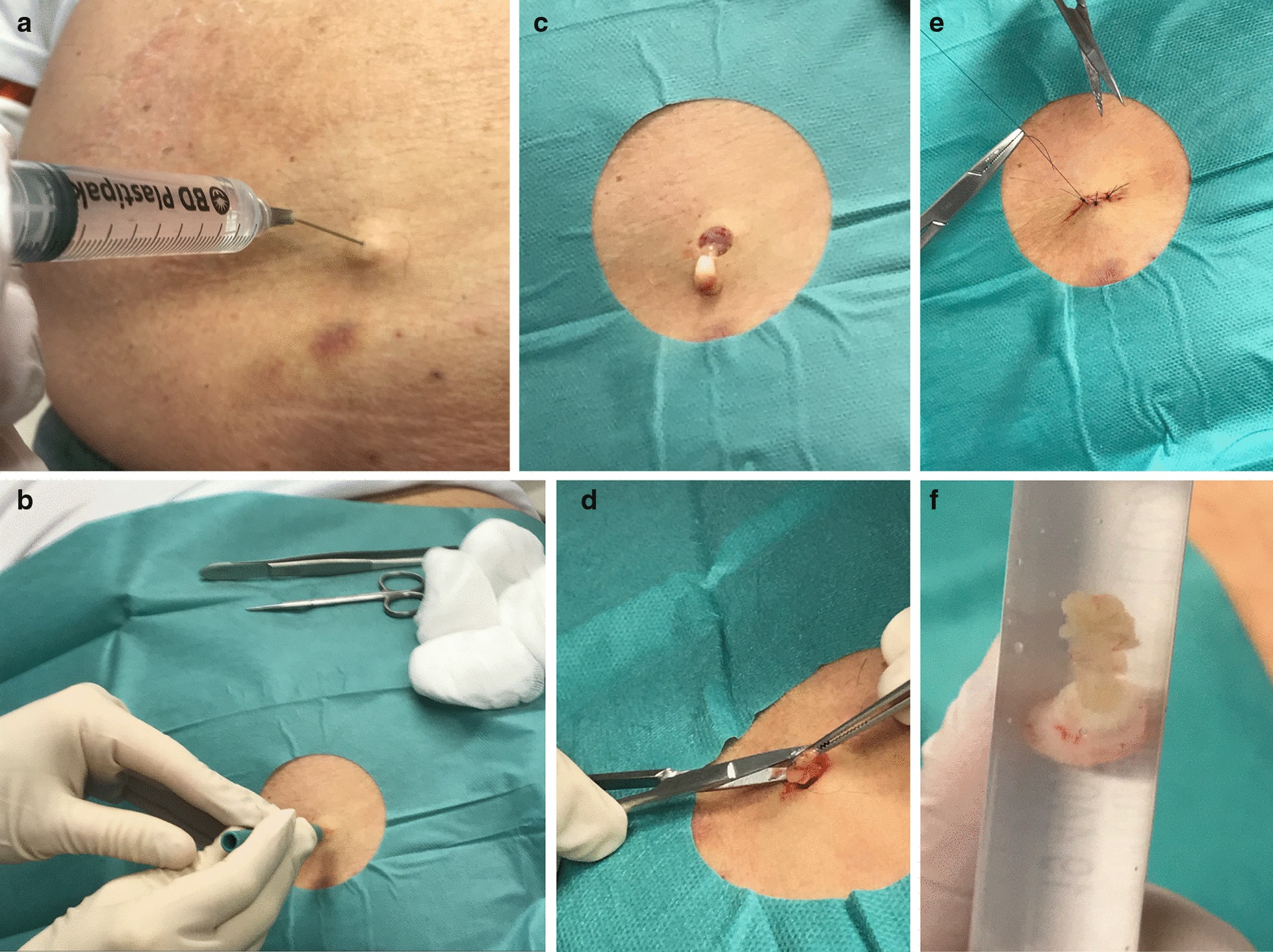
Abdominal fat pad biopsy procedure. How we perform an abdominal fat pad biopsy using an 8 mm skin punch. **a** Local anaesthesia with premixed lidocaine (10 mg/ml) with adrenaline (5 μg/ml) after disinfection with chlorhexidine (5 mg/ml). **b** Skin punch after applying a sterile drape and using sterile gloves. **c** Biopsy sample. **d** Cutting out additional fat using a pair of forceps and a pair of scissors. **e** Suture with thread size 4- 0, two simple interrupted sutures and one central vertical mattress suture is usually adequate. **f** Final biopsy sample, including skin punch and additional fat, in saline (sodium chloride 9 mg/ml)

All biopsies had been performed in local anaesthesia using an 8 mm skin punch (Fig. 1) by physicians (mainly OBS, JW and IA—co-authors) at our outpatient department as per routine clinical practice. After the diagnostic procedure, the biopsies had been stored in a saline solution and later analysed at the Department of Clinical Pathology, Uppsala University Hospital by a dedicated pathologist (PW—co-author). For amyloid diagnosis, parts of the adipose tissue (about 100 mm^3^) were put on a glass slide, cut into small pieces with a pair of scissors and squeeze preparation was obtained with the aid of another slide. The two glass slides were then dried, defatted in acetone, Congo red stained and mounted. The slides were scrutinized in a polarization microscope to determine the presence of even minute deposits of amyloid [19]. Thus, formalin-fixation, paraffin embedding and sectioning were avoided, which allowed for thicker tissue specimens to be obtained. The amyloid type was determined by immunohistochemistry or Western blot analysis as previously described [15, 20].

Patients’ medical records were reviewed and data on sex, age at disease onset, age and symptoms at the time of the biopsy, *TTR* genotype (if applicable), outcome of abdominal fat pad or any other tissue biopsies, and amyloid fibril type (if determined) were collected, as well as data from other diagnostic procedures if performed within one year from the biopsy date. Signs of peripheral polyneuropathy had been evaluated with clinical examinations at the Amyloidosis Centre, as well as with nerve conduction studies and electromyograms at the Department of Neurophysiology, Umeå University Hospital. Amyloid cardiomyopathy was considered present if ^99m^Tc-DPD scintigraphy was positive (Perugini grade 1–3) [21] and/or if the interventricular septum thickness was ≥ 12 mm on echocardiogram and no other cause of ventricular hypertrophy was evident. The scintigraphies and the echocardiograms had been performed as per local clinical practice at the Department of Radiology and at the Department of Clinical Physiology, Umeå University Hospital, respectively. ^99m^Tc-DPD scintigraphy was introduced at our centre in 2012. Gastrointestinal (GI) symptoms had been inquired for at the Amyloidosis Centre according to our routine clinical practice [22]. Signs of ocular involvement had been evaluated at the Department of Ophthalmology, Umeå University due to visual impairment or as part of our standard clinical follow-up. The patients were divided into subgroups according to their clinical symptoms/findings (polyneuropathy, cardiomyopathy, GI symptoms and eye complications, respectively).

A final diagnosis of amyloidosis had been established by the treating physician based on the clinical picture, tissue biopsy results (sometimes multiple), DNA sequencing, and in some cases, ^99m^Tc-DPD scintigraphy as per routine clinical practice. Demonstration of amyloid deposits in a tissue biopsy was considered the gold standard for diagnosis. Patients with a previously established amyloidosis diagnosis (before 2006) were excluded from further analysis, and individuals with a *TTR* mutation without any signs of ATTR amyloidosis were considered asymptomatic mutation carriers. Late onset of ATTR amyloidosis was defined as onset of symptoms after the age of 50 years as per clinical practice. *TTR* mutations are denominated using the single letter amino acid code and the sequence numbering of the mature protein.

Statistical analyses were performed with SPSS versions 25 and 26. Data are expressed as medians and full range. Specificity and sensitivity analyses were based on the outcome of the first fat pad biopsy and the established diagnosis within three years from the initial biopsy. Nominal data were analysed with chi^2^-tests. *p *values < 0.05 were regarded as statistically significant.

## Results

### Patients and diagnoses

Two hundred and seventy-six patients had undergone one or more abdominal fat pad biopsy between 2006 and 2015. Two patients were excluded due to lack of clinical data in their medical records, which resulted in 274 patients (61% males) included in the analyses (see Additional file 1). Baseline patient characteristics, at the time of the first tissue biopsy, are outlined in Table 1. All subjects had some symptom or finding suggestive of amyloidosis. No data on the time between symptom onset and the time of tissue biopsy were available.

**Table 1:** Baseline patient characteristics

	Male (n = 167)	Female (n = 107)	All (n = 274)
Age (full range)	61 (24–88) years	60 (21–86) years	60 (21–88) years
Genotype			
*TTR* mutation	105 (63%)	71 (66%)	176 (64%)
*V30M*	101 (96%)	70 (99%)	171 (97%)
*Other*	4 (4%)	1 (1%)	5 (3%)
No *TTR* mutation	26 (16%)	17 (16%)	43 (16%)
Not tested	36 (21%)	19 (18%)	55 (20%)
Symptom/finding^a^			
Polyneuropathy	146 (87%)	100 (93%)	246 (90%)
Cardiomyopathy	62 (35%)	17 (16%)	79 (29%)
GI symptoms	43 (26%)	41 (38%)	84 (31%)
Eye complications	21 (13%)	26 (24%)	47 (17%)
Other symptoms	1 (0.5%)	2 (2%)	3 (1%)
Echocardiogram			
IVSd ≥ 12 mm	68 (72%)	25 (45%)	93 (62%)
IVSd < 12 mm	26 (28%)	31 (55%)	57 (38%)
^99m^Tc-DPD scint			
Positive	18 (78%)	6 (46%)	24 (67%)
Negative	5 (22%)	7 (54%)	12 (33%)
Amyloid in first Bx	70 (42%)	39 (36%)	109 (40%)

^a^Each individual can present with one or more symptom/finding; Bx: biopsy; GI: gastrointestinal; IVSd: interventricular septum diameter; scint: scintigraphy; *TTR*: transthyretin gene. Percentages are shown as column percentage within each heading/subheading

Median follow-up after first biopsy was 8 (3–12) years. During the study period, 48 (17%) of the patients had undergone a second abdominal fat pad biopsy and 33 (12%) had been subjected to a tissue biopsy from another site (e.g. GI tract, heart, skin and kidney). Ultimately, 132 patients had got a final diagnosis of amyloidosis, mainly ATTRv amyloidosis, and 53 had been considered as asymptomatic carriers of a *TTR* mutation (Table 2). Median age at diagnosis was 63 (22–88) years. Nineteen patients (who had undergone non-diagnostic biopsies for research purposes) were excluded from further analyses due to an established amyloidosis diagnosis prior to 2006 (see Additional file 1).

**Table 2:** Final diagnoses

	Male (n = 167)	Female (n = 107)	All (n = 274)
No amyloidosis	80 (48%)	62 (58%)	142 (52%)
*TTR* mutation	22 (28%)	31 (50%)	53 (37%)
No *TTR* mutation	58 (72%)	31 (50%)	89 (63%)
Amyloidosis	87 (52%)	45 (42%)	132 (48%)
ATTRv amyloidosis	82 (94%)	41 (91%)	123 (93%)
*Dx before 2006*	10 (12%)	9 (22%)	19 (16%)
*ATTRV30M*	69 (84%)	31 (76%)	100 (81%)
*Other mutation*^a^	3 (4%)	1 (2%)	4 (3%)
ATTRwt amyloidosis	1 (1%)	0 (0%)	1 (1%)
AL amyloidosis	4 (5%)	3 (7%)	7 (5%)
*Localized*	3 (75%)	3 (100%)	6 (86%)
*Systemic*	1 (25%)	0 (0%)	1 (14%)
Gelsolin amyloidosis	0 (0%)	1 (2%)	1 (1%)

^a^H88R, A45S, V122I and A97S; AL, amyloid light chain; ATTRv, variant transthyretin amyloidosis; ATTRV30M, transthyretin amyloidosis caused by the *TTR* V30M mutation; ATTRwt, wild-type transthyretin amyloidosis; Dx, diagnosis; *TTR*, transthyretin gene. Percentages are shown as column percentage within each heading/subheading

Typing of the precursor protein (TTR) and the amyloid fibril type was available for 82 (89%) of the patients with ATTRv amyloidosis and proven amyloid deposits in their first fat pat biopsy. The majority (57%) of the patients displayed type A fibrils, and no significant difference in fibril type distribution was seen between the sexes (*p* = 0.36). Data on typing was missing in five cases, and the amount of amyloid deposits was too small for typing in five cases.

### Specificity and sensitivity

The overall test specificity was 99% and the overall sensitivity was 90% for patients with suspected systemic amyloidosis, which corresponded to positive and negative predictive values of 0.99 and 0.93, respectively. Only one false positive biopsy was found in a previously healthy 44-year-old male patient with a painful peripheral neuropathy without signs of monoclonal gammopathy and no *TTR* mutation. The first histochemical analysis showed small amounts of amyloid in the abdominal fat, but further typing was not possible. Subsequent abdominal fat pad and upper GI tract biopsies showed no signs of amyloid, which led to the conclusion that the first fat pad biopsy had been contaminated.

Due to the limited number of patients with other diagnoses, subsequent analyses were focused on the remaining 104 patients with ATTRv amyloidosis. For these patients, the results were similar with a test specificity of 99% and a sensitivity of 91% (positive and negative predictive values of 0.99 and 0.94, respectively). The initial abdominal fat pad biopsy was negative for amyloid in nine of the cases, predominantly males and late-onset patients (Table 3).

**Table 3:** Negative biopsy cases for nine patients among ATTRv amyloidosis patients diagnosed within three years from the initial biopsy

	Sex	Age	Mut	Phenotype	Onset	Fibrils	2nd fat Bx	Other Bx	Dx per
1	F	74	V30M	PN, eye	Late	NA	−	CT+	CT Bx
2	M	67	V30M	PN	Late	NA	+	GI+	Fat/GI Bx
3	M	72	V30M	PN, CM, GI	Late	NA	+	ND	Fat Bx/DPD
4	F	64	V30M	PN, GI, eye	Late	NA	ND	Skin+	Skin Bx
5	M	66	V122I	CM	Late	NA	ND	Heart+	Heart Bx
6	M	62	V30M	PN, CM	Late	Type B	+	ND	Fat Bx/DPD
7	M	58	V30M	PN, CM	Late	NA	−	ND	DPD
8	M	52	V30M	PN, CM	Late	NA	+	ND	Fat Bx/DPD
9	M	46	V30M	PN, GI	Early	NA	−	GI−	Clin. picture

Bx, biopsy; Clin, clinical; CM, cardiomyopathy; CT, carpal tunnel; DPD, ^99m^Tc-DPD scintigraphy; Dx, diagnosis; F, female; GI, gastrointestinal; M, male; Mut, mutation; NA, not available; ND, not done; PN, polyneuropathy; −, negative; +, positive

### Subgroup analyses within ATTRv amyloidosis patients

No significant differences in test sensitivity were found between the sexes (*p* = 0.34), between early and late-onset cases (*p* = 0.66) or across amyloid fibril types (*p* = 0.58).

**Fig. 2:**
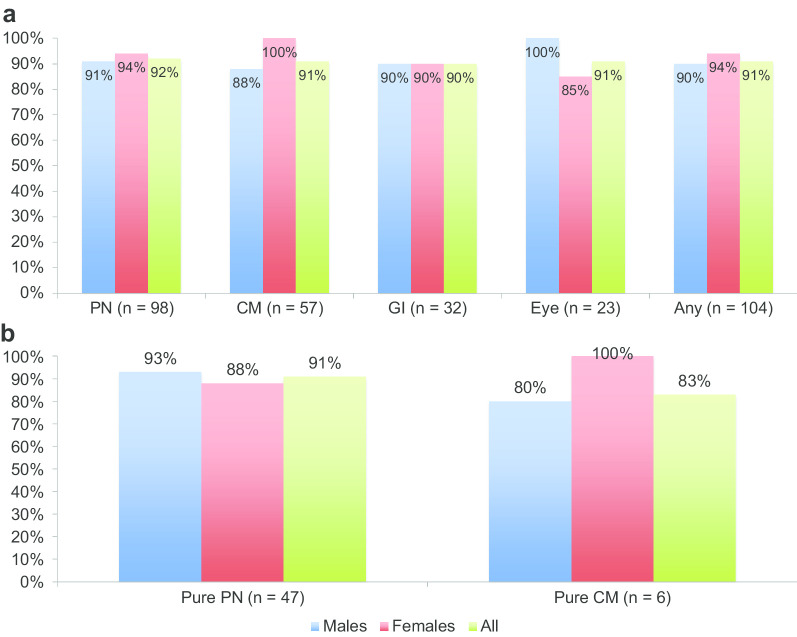
Test sensitivity in ATTRv amyloidosis patients. Sensitivity of abdominal fat pad biopsies in different subgroups of patients with a final diagnosis of hereditary transthyretin (ATTRv) amyloidosis within three years from initial biopsy. **a** Test sensitivity in subgroups as per symptom/findings and sex. Each patient can present with more than one symptom or complication, and most patients presented with a mixed phenotype. No significant differences were found between the subgroups. **b** Test sensitivity in patients with a pure neuropathic or pure cardiac phenotype. No significant differences were found between the groups. CM: cardiomyopathy; eye: eye complications; GI: gastrointestinal symptoms; PN: polyneuropathy

Among the 104 patients with ATTRv amyloidosis, the majority (94%) had signs of peripheral polyneuropathy, whereas 55% had signs of amyloid cardiomyopathy (based on echocardiograms and ^99m^Tc-DPD scintigraphies that had been performed in 89% and 27% of the patients, respectively). Most patients had a mixed phenotype with both polyneuropathy and cardiomyopathy, and only six patients (five males) had a pure cardiac phenotype at the time of the first diagnostic biopsy. Test sensitivity in relation to the patients’ sex and symptoms/findings is displayed in Fig. 2, and no significant differences were found between any of the subgroups. No negative biopsy results were found in the 15 female patients with ATTR cardiomyopathy, of whom all were late-onset cases, 13 had type A fibrils and 11 displayed a positive ^99m^Tc-DPD scintigraphy.

## Discussion

To the best of our knowledge, this is the first comprehensive evaluation of the accuracy of minimally invasive surgical fat pad biopsies for diagnosing systemic amyloidosis. The study was based on a relatively large patient material that was collected at a tertiary referral centre within the Swedish clustering area of ATTRV30M amyloidosis. Thus, the majority of patients with amyloidosis had been diagnosed with ATTRv amyloidosis, and the results should be interpreted accordingly. A clinical follow-up time of more than three years after the first diagnostic biopsy should have been sufficient to capture any false negative (or positive) biopsy results.

Overall, the diagnostic specificity and sensitivity were high (99% and 91%, respectively) and were not significantly different for any of the subgroups analysed. However, the sample sizes were too small to evaluate the diagnostic accuracy in patients with other types of amyloidosis than ATTRv amyloidosis. The number of patients was also low for some subgroups of ATTRv amyloidosis patients, especially regarding those with merely cardiac complications, which of course could have affected the outcome of the statistical analyses.

The numerically lowest sensitivity was found in male patients with ATTR cardiomyopathy. This is probably related to the amyloid fibril type since type A fibrils is associated with this phenotype, and since the type A amyloid generally is more scarcely distributed outside the clinically affected organs [13]. Six (67%) of the patients with false negative biopsies were late-onset males, and all except one had cardiomyopathy. Interestingly, no false negative biopsies were found in female patients with ATTR cardiomyopathy and type A fibrils. It is well recognised that men are more prone for cardiac ATTR deposition than women [23–27] and, thus, females may develop cardiomyopathy at later stages of the disease, which allows for more amyloid accumulation in non-cardiac tissues. Apart from this, we found no evidence of any differences in amyloid deposition in subcutaneous fat between the clinical phenotypes. However, it should be noted that patients with exclusively ocular or central nervous system (CNS) complications can present with negative peripheral tissue biopsies, although they suffer from oculoleptomeningeal ATTRv amyloidosis, since there is a local production of TTR in the eye and in the CNS.

In our material, only occasional patients with non-V30M mutations were included so no analyses of the impact of genotype on the diagnostic accuracy of abdominal fat pad biopsies were possible. Theoretically, the genotype could be of importance since it is linked to the disease phenotype and since most non-V30M mutations, as well as ATTRwt, seem to be associated with type A fibrils [28].

**Fig. 3:**
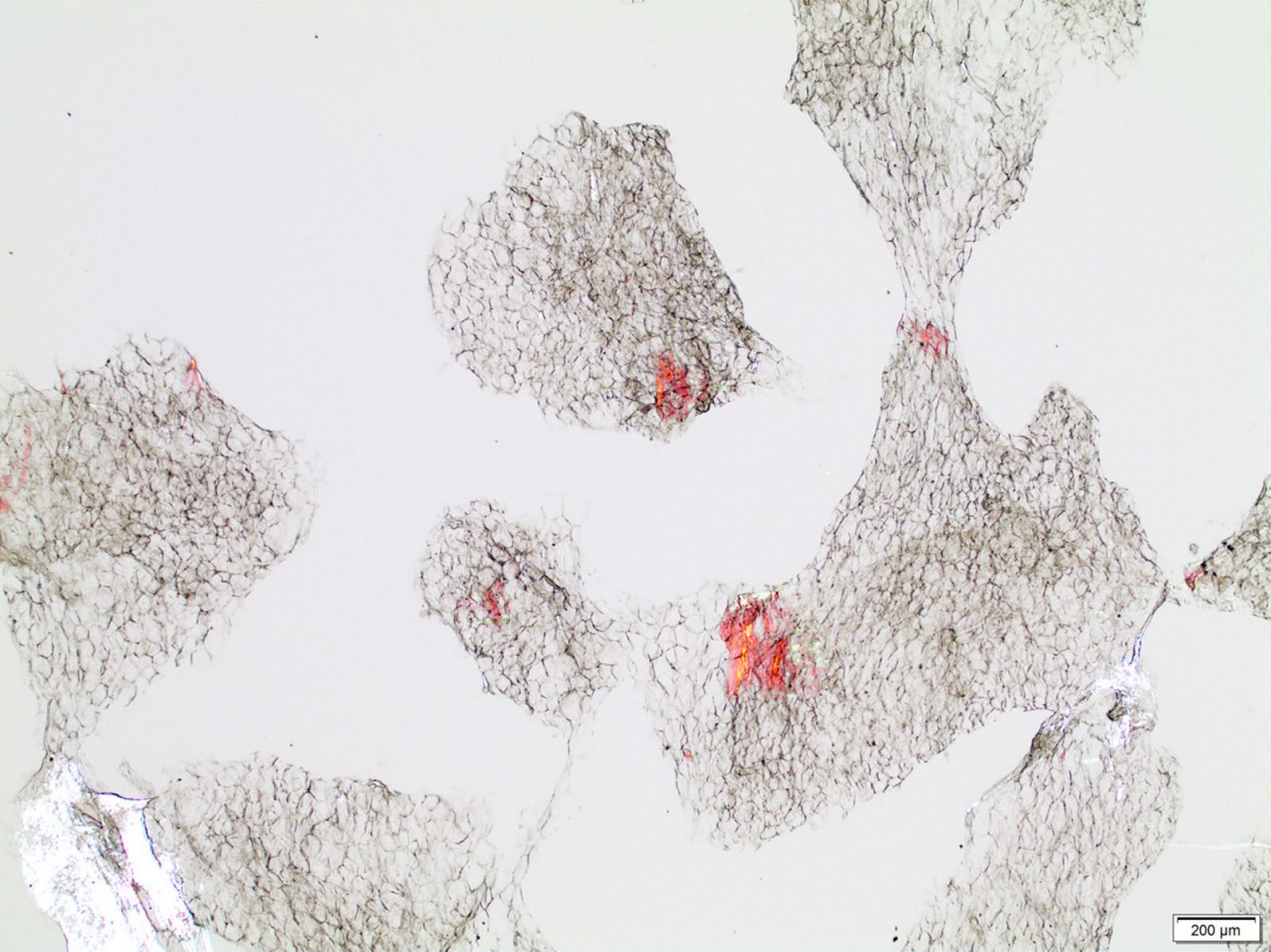
Histopathology of adipose tissue with amyloid deposits. Adipose tissue biopsy from a patient with type A transthyretin amyloid fibrils. The spotty appearance of sharply demarcated deposits is characteristic. Such deposits are sometimes small and quite few, and a systematic review is necessary. Congo red staining, normal light. Bar = 200 µm

Only one false positive fat pad biopsy was found in the study; however, it is important to note that Congo red is not an amyloid-specific dye and that it can also stain foreign particles with amyloid properties as well as collagen and hyaline [29]. This can lead to false positive results, especially if the tissue sample is over stained and if the specimen is analysed at a laboratory with limited experience of amyloid diagnosis. An advantage of subcutaneous adipose tissue in this context is that it contains low levels of proteins compared to other tissues [15]. With the method used in the present study, the preparations are thicker compared to those generally used for microscopy, and Congo red stained deposits thereby appear more clearly and are more easily distinguishable from the background (Fig. 3). It is also our experience that ATTR amyloid, particularly of type A, shows a stronger stainability with Congo red if formalin fixation and paraffin embedment are avoided.

Nonetheless, contamination of the sample with for example textile fibres can be interpreted as amyloid deposits, which was probably the case in our patient; therefore, it is important to minimize the use of textile tissues and swabs during the biopsy procedure. Moreover, long-term subcutaneous injections of insulin due to diabetes mellitus can give rise to local insulin-derived amyloid deposits [30], which is of course important to be aware of when performing an abdominal fat pad biopsy for amyloid diagnosis on a patient with insulin treatment.

With the above in mind, it is evident that typing of the amyloid is central for verification of the precursor protein. We have developed in-house antibodies to use for typing with immunohistochemistry or Western blot, since commercial antibodies often fail to determine ATTR fragments [15, 20]. However, proteomics and mass spectrometry have also been shown to be feasible alternatives [31]. From our experience, an abdominal fat pad biopsy using a skin punch is a safe and convenient way to obtain an adequate tissue sample that enables reliable detection of amyloid, as well as determination of the precursor protein and of the ATTR fibril type. In the current study, typing was possible in the majority of patients although the amount of amyloid was too small for determining the precursor protein and the fibril type in five cases. Typing with Western blot is usually not possible with biopsies from other organs, since the amount of tissue sent for histopathology is smaller and thereby also is the quantity of amyloid. However, mass spectrometry can be an alternative method for amyloid typing in these cases [32].

Currently at our centre, we primarily use surgically obtained abdominal fat pad biopsies for diagnosing systemic amyloidosis. We previously performed needle aspiration (using 16–20 Gauge needles) of abdominal fat after local anaesthesia, but have discarded this procedure since it was painful for the patient and difficult to obtain a sufficient amount of tissue for a reliable histopathological evaluation. In addition, blood contamination of the sample was a problem. However, good sensitivity has been reported utilising this method if a sufficient amount of fat can be obtained [33]. The diagnostic sensitivity of fine needle aspirates was shown to be unreasonably low for patients with ATTR amyloidosis, especially for patients with ATTR cardiomyopathy [16, 17, 34]. Although skin punch biopsies are slightly more invasive than needle aspiration, we have not experienced a higher rate of complications with this technique. Therefore, we strongly suggest using skin punch biopsies, rather than fine needle aspiration, to obtain fatty tissue samples in patients with suspected ATTR amyloidosis.

Although the diagnostic sensitivity of the abdominal fat pad biopsies was high in our material, repeated fat pad biopsies and/or alternative biopsy sites were required to establish the diagnosis in some cases. This is probably difficult to avoid since the amyloid deposits can be scarce in early stages of the disease and also unevenly distributed in the tissue, especially in patients with type A ATTR fibrils [16, 35]. It is also possible that ATTR amyloidosis has developed during the time between biopsies.

If amyloidosis is strongly suspected and the abdominal fat pad biopsy is negative for amyloid, we usually repeat the biopsy after six months or plan for a biopsy from another organ (e.g. GI tract or heart) depending on the clinical picture. The diagnostic accuracy for other commonly used biopsy sites, such as sural nerve, GI tract and labial salivary glands, is varying. Sural nerve biopsy has a reported test sensitivity of 73–87% [36–38]; however, the procedure induces permanent nerve damage and is not a preferred site for diagnostic biopsies at our centre. GI tract biopsies was reported to have an average diagnostic sensitivity of 16% in a large retrospective study from Germany; however, the sensitivity increased to 99% if submucosal vessels were included in the biopsies [39]. Another retrospective analysis from the Mayo Clinic reported a diagnostic sensitivity of 81% for rectal biopsies in ATTR cardiac amyloidosis [16]. We occasionally perform gastroduodenal or rectal biopsies for diagnostic purposes at our centre. Labial salivary glands is the preferred biopsy site at many centres and has a reported sensitivity of 75% to 91%, the latter achieved in a Portuguese population of generally early onset ATTRV30M patients [40, 41]. We have no experience of this procedure but it is an acceptable diagnostic option, although it appears to be more uncomfortable for the patients than abdominal fat pad biopsies. Unlike adipose tissue biopsies, all of the abovementioned techniques require fixation, paraffin-embedding and sectioning of the tissue. By avoiding these steps, type A ATTR is more strongly stained with Congo red, and small amyloid deposits are more easily identified in the thicker (up to 100 μm) adipose tissue fragments obtained with our method. In the case of suspect ATTR cardiomyopathy, bone scintigraphy can be a good alternative to a tissue biopsy as long as a monoclonal gammopathy has been ruled out [18]. However, it should be noted that the sensitivity for bone scintigraphy is low for patients with type B ATTR fibrils [42].

In Sweden, we normally require demonstration of amyloid deposits in a tissue biopsy to establish the diagnosis of ATTR amyloidosis, even in patients with a proven *TTR* mutation and typical symptoms of amyloidosis. This is due to the low penetrance of ATTRv amyloidosis in the Swedish population [43], and due to our rather old patient population with other potential causes of both polyneuropathy and heart disease. Therefore, tissue biopsies are liberally performed, especially in regard to *TTR* mutation carriers. Since the ATTR fibril type is associated with the disease phenotype and with the survival after liver transplantation [13, 14], we also find it essential to try to establish the amyloid fibril type. It remains to be elucidated whether the amyloid fibril type also has any implications on the response to other therapies, such as TTR stabilisers and TTR gene silencing.

## Conclusions

Abdominal fat pad biopsies performed with an 8 mm skin punch exhibit good diagnostic accuracy in patients with suspect ATTRv amyloidosis when histopathology is performed at a specialized centre. Further, test sensitivity was not significantly lower for patients with cardiomyopathy or for other subgroups of patients. We therefore conclude that abdominal fat pad biopsies is a safe and reliable diagnostic tool in ATTRv amyloidosis that enables typing of the precursor protein and of the amyloid fibril type.

## Supplementary information

Additional file 1. Patient flow chart. Data were evaluated from all patients who had undergone at least one abdominal fat pad biopsy between January 2006 and December 2015. A final diagnosis of amyloidosis had been established by the treating physician based on the clinical picture, tissue biopsy results, DNA sequencing, and in some cases, 99mTc-DPD scintigraphy as per routine clinical practice. Patients with a previously established amyloidosis diagnosis (before 2006) were excluded from further analysis. ATTRv: variant transthyretin amyloidosis; ATTRV30M: transthyretin amyloidosis caused by the TTR V30M mutation; TTR: transthyretin gene.

## Data Availability

The datasets used and/or analysed during the current study are available from the corresponding author on reasonable request.
