# Supplementary figures and images for: Abdominal fat pad biopsies exhibit good diagnostic accuracy in patients with suspected transthyretin amyloidosis
Source: Orphanet J Rare Dis. 2020 Oct 8;15:278. doi: 10.1186/s13023-020-01565-8 (PMC7545559; doi:10.1186/s13023-020-01565-8)

## Slide 1
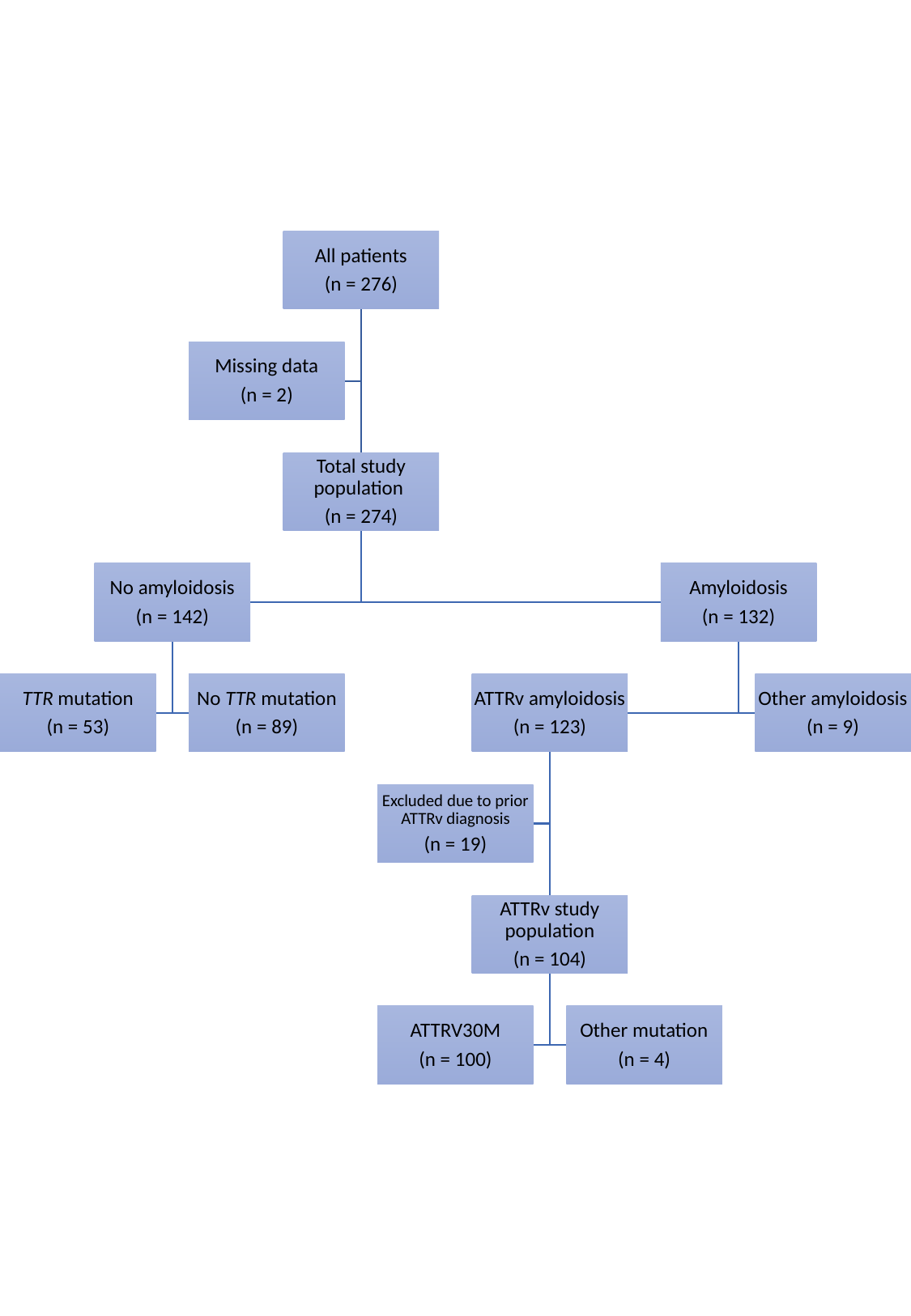

Supplement: Supplementary file 1 — Patient flow chart. Data were evaluated from all patients who had undergone at least one abdominal fat pad biopsy between January 2006 and December 2015. A final diagnosis of amyloidosis had been established by the treating physician based on the clinical picture, tissue biopsy results, DNA sequencing, and in some cases, 99mTc-DPD scintigraphy as per routine clinical practice. Patients with a previously established amyloidosis diagnosis (before 2006) were excluded from further analysis. ATTRv: variant transthyretin amyloidosis; ATTRV30M: transthyretin amyloidosis caused by the TTR V30M mutation; TTR: transthyretin gene. [file 13023_2020_1565_MOESM1_ESM.pptx]
